# Micro Fault Diagnosis of Driving Motor Bearings Based on Multi-Residual Neural Networks and Evidence Reasoning Rule

**DOI:** 10.3390/e28010053

**Published:** 2025-12-31

**Authors:** Aoxiang Zhang, Lihong Tang, Guanyu Hu

**Affiliations:** Key Laboratory of the Ministry of Education, Guilin University of Electronic Technology, Guilin 541004, China; zhangaoxiang9469@163.com (A.Z.); 24032306030@mails.guet.edu.cn (L.T.)

**Keywords:** micro-fault diagnosis, multi-residual neural network, ER Rule, diagnostic condition assessment

## Abstract

Micro-fault diagnosis of vehicle driving motor bearings can significantly bring safety and economic benefits in preventing major accidents and extending equipment lifespan. However, under variable operating conditions, effectively capturing and diagnosing fault-related weak current fluctuation or high-frequency noise features, presents substantial technical challenges. Regarding these issues, this paper proposes multi-residual neural networks (multi-ResNets) and an evidential reasoning rule (ER Rule)-based fault diagnosis model. The model employs a benchmark condition generalization mechanism, which selects multiple typical load conditions as diagnostic anchor points based on a multi-residual neural network structure. Furthermore, by integrating a sub-model credibility assessment mechanism to perform diagnostic condition assessment and category assessment based on ER rule. The experimental results indicate that compared to the traditional machine learning algorithms, the proposed multi-ResNets-ER Rule-based model achieves higher diagnostic accuracy and result reliability for micro-faults under variable operating conditions.

## 1. Introduction

MICRO-faults as a typical fault type in vehicle driving motor bearings, due to their strong concealment and rapid development, if not addressed in time, micro-faults can trigger a chain reaction of bearing failure, leading to major safety incidents such as motor burnout or vehicle loss of control. When early detected and diagnosed, it can not only eliminate risks at the bud, enhancing vehicle operational safety, but also promotes the traditional “periodic maintenance” model to upgrade to an intelligent “condition monitoring + predictive maintenance” model. This optimizes bearing maintenance cycles, reduces resource waste caused by over-maintenance, and lowers overall vehicle operating costs.

However, due to the concealment and randomness of minor faults, their characteristics are often very difficult to detect. Moreover, fault signatures are more prone to being masked by interference or noise caused by vehicle operating conditions and load changes, making early monitoring and fault localization particularly challenging.

These issues impose higher demands on diagnostic technologies in terms of feature extraction, diagnostic accuracy, and reliability. Micro-fault diagnosis is not only one of the core challenges in the current field of fault diagnosis, but also an urgent problem that needs to be solved.

By organizing and analyzing current investigations, the research on micro-fault diagnosis mainly includes the following three types.

Knowledge-based approach: The diagnostic method based on qualitative knowledge is suitable for fields with complex system structures and extensive historical fault data, offering strong explainability and reasoning capabilities.

For the fault diagnosis and monitoring of gas turbine combustion chamber, ref. [1] presents the fundamental working principles, conceptual design framework, and development methodology of a gas turbine combustion chamber expert system. In addressing energy system fault diagnosis challenges, ref. [2] proposes an innovative approach that integrates evolutionary algorithm recombination with artificial random selection operator generation to construct the rule-based knowledge base of expert systems. For process industry safety protection applications, ref. [3] develops a knowledge-based fault diagnosis methodology that employs fuzzy logic to synergistically process expert domain knowledge and real-time operational data, culminating in the creation of a computer-aided diagnostic tool. In the domain of automotive fault detection, ref. [4] introduces an expert system (ES)-based framework for electric vehicle fault modeling, implementing the resulting Car Fault Diagnosis Auxiliary System (CFMDAS). To overcome inherent limitations of conventional expert systems regarding generality and expandability, ref. [5] proposes a task-oriented software architecture that enables context-specific optimization through a configurable rule set while maintaining user accessibility for rule modification via human-machine interfaces, thereby facilitating continuous professional knowledge acquisition. Finally, ref. [6] establishes a comprehensive fault diagnosis expert system framework characterized by two core components: a hierarchical diagnostic strategy and a knowledge base incorporating fault classification and unit-under-test detection methodologies. Ref. [7] proposes an automatic interval belief rule base (A-IBRB) method to address expert knowledge acquisition challenges. The model integrates error-constrained k-means++, Gaussian interval belief initialization, and evidential reasoning, optimized by a projected CMA-ES algorithm. Gearbox and bearing fault diagnosis cases validate its effectiveness and reliability.

However, under variable operating conditions, the difficulty in extracting specific micro-fault features and expressing knowledge-based rules limits their diagnostic applicability in such scenarios.

Analytical model-based approach: The analytical diagnosis model is built upon a deep understanding of system fault evolution mechanisms, involving high-precision mathematical modeling of the system. It offers advantages such as high real-time performance and robustness in diagnosis.

In ref. [8], a hierarchical multi-model fault diagnosis scheme utilizing the Unscented Kalman Filter (UKF) is proposed for precise detection and isolation of actuator faults in robotic systems. Building upon this, ref. [9] extends the Observer-based Localization Algorithm (OLA) from continuous systems to nonlinear discrete systems, providing comprehensive stability analysis and robustness evaluation of both fault detection and compensation algorithms. Specifically addressing four-rotor actuator faults, ref. [10] designs an integrated fault diagnosis system incorporating a nonlinear fault detection estimator and a nonlinear adaptive fault isolation estimator, demonstrating significant improvements in system robustness and algorithm sensitivity. Furthermore, ref. [11] develops a robust navigation-based fault diagnosis system for underwater robotic platforms through particle filtering techniques, achieving effective fault identification while maintaining superior operational robustness.

For the diagnosis of micro-fault under variable operating conditions, a profound understanding of fault evolution mechanisms and precise mathematical modeling prove highly challenging. Moreover, the subtle nature of micro-faults makes them easily buried in noise, necessitating models with exceptional robustness and fault sensitivity.

Data-driven approach: Traditional data-driven fault diagnosis algorithms directly establish input-output mapping relationships by leveraging noise reduction and feature extraction algorithms. This enables them to model complex nonlinear systems.

In ref. [12], a discrete inverse wavelet transform methodology is employed to extract pertinent frequency bands from operational data, enabling the reconstruction and filtering of current sequences under distinct fault conditions. Subsequently, trend fluctuation analysis is conducted to achieve effective differentiation of minor faults. Building upon this, ref. [13] systematically reviews the evolution and application of the continuous wavelet transform (CWT) in rolling bearing micro-fault diagnosis, introducing a decision tree framework for optimal wavelet selection that enhances classification accuracy. Furthermore, ref. [14] proposes an advanced auditory signal fault diagnosis technique by synergistically integrating wavelet transform with empirical mode decomposition (EMD), leveraging sample entropy and singular value decomposition (SVD) to reduce signal-to-noise ratio while improving SVD performance. To address component degradation and mechanical wear in production systems, ref. [15] implements an artificial neural network (ANN)-based approach for minor fault detection and diagnosis through time-frequency analysis. Finally, ref. [16] designs a novel continuous decision function for support vector machine (SVM) classifiers, enabling simultaneous fault type identification and severity monitoring.

The data-driven fault diagnosis model has achieved promising results in detecting micro-faults. However, its generalization capability remains limited—when operating conditions (e.g., load or environmental variations) change, the model’s performance may degrade significantly.

To address the challenges of mechanical micro-fault diagnosis under variable operating conditions, this paper proposes a fault diagnosis model based on a multi-residual neural network structure and ER rule.

First, a benchmark condition generalization mechanism was employed within model, which selects multiple typical load condition as diagnostic anchor points based on a multi-residual neural network structure. For each typical load conditions, an independently trained deep learning sub-model was adopted to extract micro-fault features and perform diagnosis reasoning. Compared to the traditional artificial intelligence algorithms, deep learning models [17,18,19,20,21,22,23,24,25,26] can pursue fundamental data characteristics and structural information at a deeper level.

Furthermore, based on a sub-model credibility assessment mechanism, the cross-entropy loss function is used to calculate the sub-model divergence between predicted probability distribution and target probability distribution, and by using a predefined credibility assessment formula, the loss value of each sub-model further converted into relative credibility value, while also serves as belief degree for diagnostic condition assessment and serves as the weight in subsequent diagnostic category assessment.

Case studies demonstrate that compared to traditional machine learning algorithms, the proposed fault diagnosis model achieves higher diagnostic accuracy and result reliability for micro-fault issues under varying operational conditions.

The paper is organized as follows. Section 2 elaborates on the theoretical basis. Section 3 proposes a case study for performance verification and result analysis. Conclusions are explained in Section 4.

## 2. The Theoretical Basis

### 2.1. Information Transformation

Residual neural network model should utilize the micro-fault images for feature extraction, as the time-frequency analysis tool, the short-time Fourier transform (STFT) [27,28] can generate continuous spectrum through the time-frequency transformation of non-stationary signals:

(1) $$G_{f}\left(\varepsilon ,u\right)=\int f\left(t\right)g\left(t-u\right)e^{j\varepsilon t}dt$$

where f(t) is the original signal and g(t−u) is the window function.

### 2.2. Training and Reasoning of Residual Neural Networks

**Figure 1:**
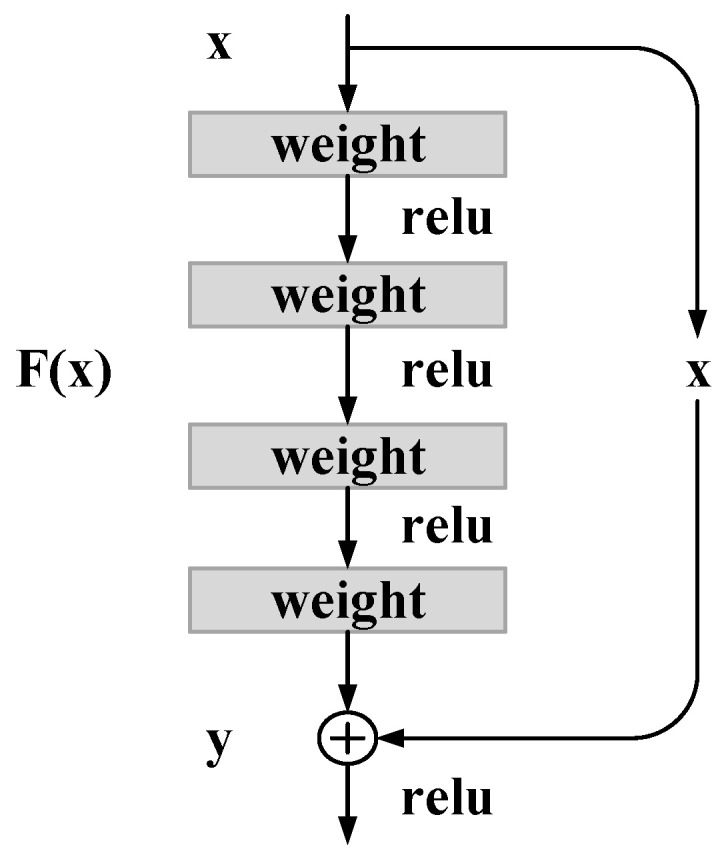
Residual module of the residual neural network.

Residual module as the basic building block of the residual neural network, in this section, a four-layer residual module (see Figure 1) serves as an example to illustrate the forward and backward propagation processes, with the corresponding equations provided below:

(2) $$F\left(x,\left\{W\right\}\right)=W_{4}\sigma \left(W_{3}\sigma \left(W_{2}\sigma \left(W_{1}x+b_{1}\right)+b_{2}\right)+b_{3}\right)$$

(3) y=σ(F(x,{W})+x)

where *x* directly imports from the topmost layer; σ represents the activation function, *W* represents the convolution kernel weight, and *b* represents the bias. *y* is the output vector of the residual module.

For the output of the residual neural network, the cross-entropy loss function is adopted as follows:

(4) $$\mathrm{softmax}\left(y\right)=\frac{e^{y}}{\sum e^{y}}$$

(5) $$\mathrm{Loss}=-\sum \mathrm{softmax}\left(y\right)\log y_{\mathrm{label}}$$

where softmax(y) is used for normalization; $y_{\mathrm{label}}$ represents the target output; Loss represents the loss value.

Based on the chain rule, the partial derivative of the loss function value can be obtained.

(6) $$\frac{\partial \mathrm{Loss}}{\partial y}=y-y_{\mathrm{label}}$$

(7) $$\frac{\partial \mathrm{Loss}}{\partial W_{4}}=\frac{\partial \mathrm{Loss}}{\partial y}\frac{\partial y}{\partial (F(x,\{W\})+x)}\frac{\partial (F(x,\{W\})+x)}{\partial W_{4}}$$

(8) $$\frac{\partial \mathrm{Loss}}{\partial W_{3}}=\frac{\partial \mathrm{Loss}}{\partial y}\frac{\partial y}{\partial (F(x,\{W\})+x)}\frac{\partial (F(x,\{W\})+x)}{\partial y_{3}}\frac{\partial y_{3}}{\partial W_{3}}$$

(9) $$\frac{\partial \mathrm{Loss}}{\partial W_{2}}=\frac{\partial \mathrm{Loss}}{\partial y}\frac{\partial y}{\partial (F(x,\{W\})+x)}\frac{\partial (F(x,\{W\})+x)}{\partial y_{3}}\frac{\partial y_{3}}{\partial y_{2}}\frac{\partial y_{2}}{\partial W_{2}}$$

(10) $$\frac{\partial \mathrm{Loss}}{\partial W_{1}}=\frac{\partial \mathrm{Loss}}{\partial y}\frac{\partial y}{\partial (F(x,\{W\})+x)}\frac{\partial (F(x,\{W\})+x)}{\partial y_{3}}\frac{\partial y_{3}}{\partial y_{1}}\frac{\partial y_{1}}{\partial W_{1}}$$

(11) $$W:=W-\alpha \frac{\partial \mathrm{Loss}}{\partial W}$$

where *W* represents the neural network’s original weight, α represents the learning rate, and *W* represents the update weight.

### 2.3. Diagnostic Condition Assessment

Cross-entropy loss function reflects the deviation between the predicted probability distribution and the target probability distribution. In this subsection, the loss value is used to measure the relative credibility among the sub-models and serves as a belief degree for diagnostic condition assessment, while also serving as the weight for subsequent diagnostic category assessment:

(12) $$Loss_{i}=-\sum_{n=1}^{N}\beta_{n,i}\log\left(\beta_{n,i}^{\prime }\right),\left(n=1,2,\ldots ,N\right)$$

(13) $$C_{i}=\frac{\ln Loss_{i}}{\sum_{i}^{L}\ln Loss_{i}},\sum_{i}^{L}C_{i}=1,0\leq C\leq 1\left(i=1,2,\ldots ,L\right)$$

(14) $$C_{i}=\beta_{Hi},C_{i}=\omega_{i},i=1,2,\ldots ,L$$

(15) $$S\left(H_{U}\right)=\left\{\left(H_{i},\beta_{Hi}\right),i=1,2,\ldots ,L\right\}$$

(16) $$H_{U}=\sum_{i=1}^{L}H_{i}\beta_{Hi}$$

where $\beta_{n,i}$ represents the target category probability in one-hot encoded form, $\beta_{n,i}^{\prime }$ is the $i^{th}$ sub-model’s predicted category probability of the $n^{th}$ category after normalization, $C_{i}$ represents the relative credibility among the sub-models, $S(H_{U})$ represents the belief distribution, $\beta_{Hi}$ represents the belief degree of diagnostic condition $H_{U}$ relative to the condition anchor point $H_{i}$, and $\omega_{i}$ represents $i^{th}$ sub-model’s weight for subsequent diagnostic category assessment.

### 2.4. Diagnostic Category Assessment

The proposed model uses Evidence Reasoning Rule (ER Rule) as the diagnostic reasoning fusion theory. First, the probability distributions of the diagnostic reasoning corresponding category need to be combined with the corresponding weights $\omega_{i}$ to obtain the basic probability masses.

(17) $$m_{n,i}=\omega_{i}\beta_{n,i},\sum_{i=1}^{L}\omega_{i}=1,0\leq \omega_{i}\leq 1\;n=1,2,\ldots ,N$$

(18) $$m_{H,i}=1-\sum_{n=1}^{N}m_{n,i}=1-\omega \sum_{n=1}^{N}\beta_{n,i}$$

where $m_{n,i}$ represents the basic probability masses. $m_{H,i}$ indicates the remaining basic probability masses which can be broken down into ${\bar{m}}_{H,i}$ and ${\tilde{m}}_{H,i}$ two main parts.

(19) $${\bar{m}}_{H,i}=1-\omega_{i}$$

(20) $${\tilde{m}}_{H,i}=\omega_{i}\left(1-\sum_{n=1}^{N}\beta_{n,i}\right)$$

Second, by using the basic probability masses, the *i* diagnostic reasoning and the (i+1)th diagnostic reasoning can be aggregated into a new diagnostic reasoning, the aggregation process as follows:

(21a) $$\left\{H_{n}\right\}:\;m_{n,I(i+4)}=K_{I(i+1)}\left[m_{n,I(i)}m_{n,i+1}+m_{H,I(i)}m_{n,i+1}+m_{n,I(i)}m_{H,i+1}\right]$$

(21b) $$m_{H,I(i)}={\tilde{m}}_{H,I(i)}+{\bar{m}}_{H,I(i)},\;n=1,2,\ldots ,N$$

(21c) $$\left\{H_{n}\right\}:\;{\tilde{m}}_{H,I(i+1)}=K_{I(i+1)}\left[{\tilde{m}}_{H,I(i)}{\tilde{m}}_{H,i+1}+{\bar{m}}_{H,I(i)}{\tilde{m}}_{H,i+1}+{\tilde{m}}_{H,I(i)}{\bar{m}}_{H,i+1}\right]$$

(21d) $$\left\{H_{n}\right\}:\;{\bar{m}}_{H,I(i+1)}=K_{I(i+1)}\left[{\bar{m}}_{H,I(i)}{\bar{m}}_{H,i+1}\right]$$

(21e) $$K_{I(i+1)}={\left[1-\sum_{i=1}^{N}\sum_{j=1,j\neq i}^{N}m_{i,I(i)}m_{j,i+1}\right]}^{-1},\;i=\left\{1,2,\ldots ,L-1\right\}$$

where $m_{n,I(i+1)}$ is the combined probability mass generated by aggregating the first *i* diagnostic reasoning with the (i+1)th diagnostic reasoning; ${\tilde{m}}_{H,I(i+1)}$ the combined probability mass for due to the possible incompleteness, and ${\bar{m}}_{H,I(i+1)}$ for due to the combined relative importance.

Finally, after all *L* diagnostic reasoning have been aggregated, the combined probability distribution are generated by using the following normalization process:

(22) $$\left\{H_{n}\right\}:\;\beta_{n}=\frac{m_{n,I(L)}}{1-{\bar{m}}_{H,I(L)}},\;n=1,2,\ldots ,N$$

(23) $$S\left(y_{fault}\right)=\left\{\left(y_{fault_{n}},\beta_{n}\right),n=1,2,\ldots ,N\right\}$$

where $\beta_{n}$ is the diagnostic model’s diagnostic category probability of the $n^{th}$ fault category $y_{fault_{n}}$ after normalization.

## 3. Performance Verification and Result Analysis

### 3.1. Case Description and Model Construction

In this subsection, the CWRU bearing dataset is selected to verify the model’s ability. The dataset is one of the most widely used datasets in the field of bearing fault diagnosis, and the motor bearing and load configuration closely match the dynamic operating conditions of vehicle driving motor, which effectively simulating key characteristics such as load switching and speed fluctuations.

It covers four load conditions of 0, 1, 2, and 3 horsepower, and includes three fault types: normal condition, inner race fault, outer race fault, and rolling element fault, with fault diameters categorized into three specifications: 7 mils, 14 mils, and 21 mils.

**Figure 2:**
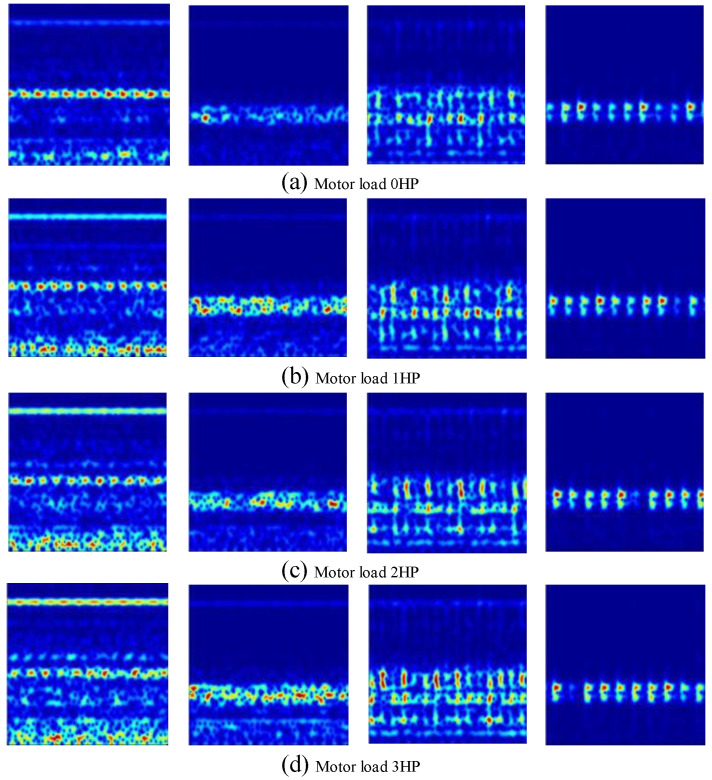
The defect spectrum of the samples under four conditions.

For the mechanical micro-fault diagnosis under variable operating conditions, the drive end bearing under the minimum defect size (7 mils) is selected as the diagnostic object, and the drive end data, sampling frequency at 48 kHz, are used to establish the dataset. For each bearing, faults are defected under each load condition, including 800 training samples and 200 test samples. Table 1 provides the drive-end bearing faults under four load conditions. Figure 2 shows the sample’s defect spectrum generated by STFT, with a size of 224 × 224 × 3.

**Figure 3:**
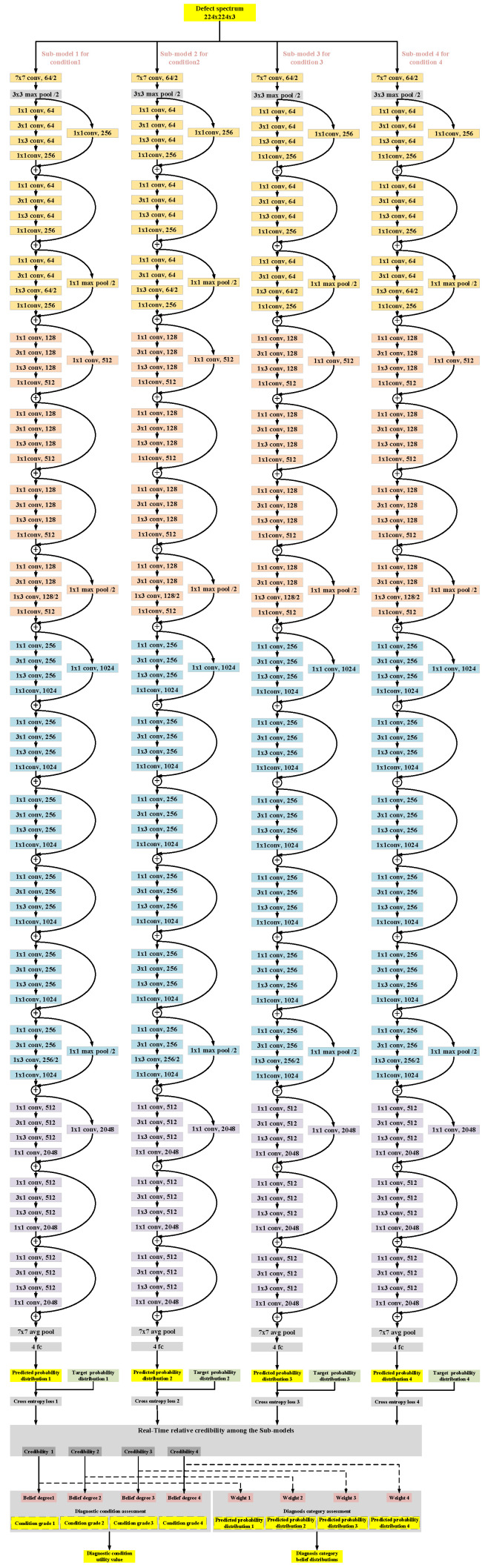
The structure of diagnostic model based on ResNet network and ER rule.

For this case, a diagnostic model was established as shown in Figure 3, and the detailed structural parameters are given in Table 2. It consists of two main parts: the diagnostic reasoning part and the diagnostic assessment part. The diagnostic assessment part further includes three modules: the sub-models credibility assessment, the diagnostic condition assessment, and the diagnostic category assessment.

To ensure the adaptability of the diagnostic model in vehicle edge computing scenarios, four lightweight 67-layer residual neural network models were employed within the diagnostic reasoning part, and for each load condition, an independent sub-model is employed for training and diagnostic reasoning. Finally, the diagnostic condition assessment and diagnostic category assessment are conducted based on the ER rule.

### 3.2. Model Training and Result Analysis

**Figure 4:**
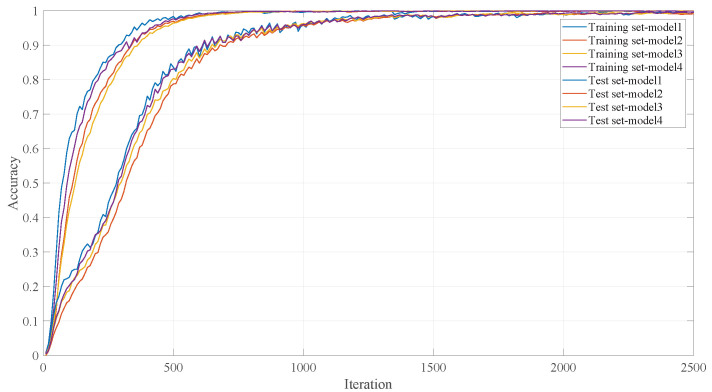
The correct rate of the Resnet-67 model-1∼4.

**Figure 5:**
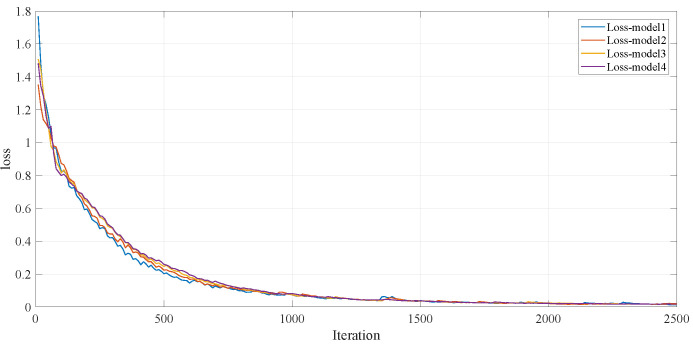
The loss value of the Resnet-67 model-1∼4.

In this subsection, the model conducted 2500-round iterative training, and the training results are shown in Figure 4 and Figure 5. Wherein Figure 4 shows the correct rate changes of the ResNet-67 model-1 4 separately on the corresponding training set and test set. The change curves show that the sub-models have a high accuracy on the corresponding training set and test set, and there is no obvious overfitting phenomenon.

Figure 5 shows the loss value change of ResNet-67 model-1∼4 separately on the corresponding training set, according to the change curves of loss values, sub-models have the ability of rapid optimization.

**Figure 6:**
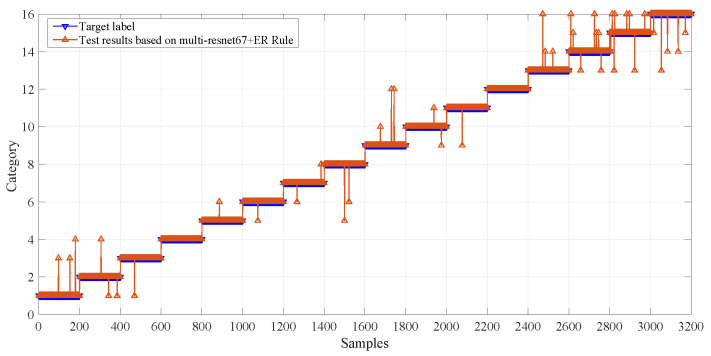
The diagnostic reasoning of the diagnostic model on the test set.

Figure 6 shows the diagnostic reasoning of the multi-ResNet-67-ER rule-based model on the test set. The diagnostic model has a high identification accuracy for all diagnostic categories under four working conditions.

### 3.3. Experimental Comparison

**Figure 7:**
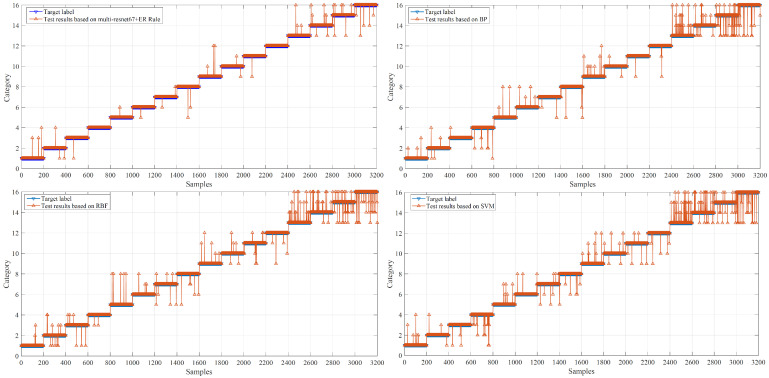
The diagnostic reasoning results of the multi-ResNet-67-ER rule model and traditional models.

To evaluate the model’s performance, this subsection presents traditional models based on the BP neural network [29], RBF neural network [30], and SVM [31] for comparative evaluation. All four fault categories under four load conditions are treated as independent discrete classes during traditional models training (totaling 16 classes). The correct rate on the test set between the proposed model and traditional models is shown in Figure 7 and summarized in Table 3.

The former presents the models’ diagnostic reasoning results, while the latter displays the correct rates on the training set and test set. The proposed model achieves a higher correct rate than those of the BP network, RBF network, and SVM models. These comparisons demonstrate the superior performance of the model.

## 4. Conclusions

To tackle the micro-fault diagnosis of the vehicle power train system under variable operating conditions, this paper introduces a multi-ResNet67 and ER rule-based fault diagnosis model. The model employs a benchmark condition generalization mechanism, which selects multiple typical load conditions as diagnostic anchor points and integrates a sub-model credibility assessment mechanism to perform diagnostic condition assessment and category assessment. This integration achieves the model’s micro-fault diagnosis under varying vehicle operating conditions. Case studies demonstrated that, compared to traditional machine learning algorithms, the proposed model achieves higher diagnostic accuracy for micro-faults under variable operating conditions.

The contributions of this study can be summarized as follows:

1. A diagnostic model employing a benchmark condition generalization mechanism was proposed, which selects multiple typical load conditions as diagnostic anchor points based on a multi-residual neural network structure.
2. By integrating a sub-model credibility assessment mechanism to perform diagnostic condition assessment and category assessment based on ER rule, this model achieves micro-fault diagnosis under varying vehicle operating conditions.

## Figures and Tables

**Table 1:** The bearing faults defect under four load conditions.

Motor Load (HP)	Motor Speed (rpm)	Normal	Inner Raceway	Ball	Outer Raceway Center
0	1797	-	7 mils	7 mils	7 mils
1	1772	-	7 mils	7 mils	7 mils
2	1750	-	7 mils	7 mils	7 mils
3	1730	-	7 mils	7 mils	7 mils

**Table 2:** The parameters of the diagnostic model.

Residual Unit	Output Size	Network Layer Parameters	Unit Number	Sub-Model Number
-	112 × 112	7 × 7 conv, 64/2	1	4
-	56 × 56	3 × 3 max pool, 64/2	1	
unit_1	56 × 56	1 × 1, 64; 3 × 1, 64; 1 × 3, 64; 1 × 1, 256	2	
unit_1	56 × 56	1 × 1, 64; 3 × 1, 64; 1 × 3, 64/2; 1 × 1, 256	1	
unit_2	28 × 28	1 × 1, 128; 3 × 1, 128; 1 × 3, 128; 1 × 1, 512	3	
unit_2	28 × 28	1 × 1, 128; 3 × 1, 128; 1 × 3, 128/2; 1 × 1, 512	1	
unit_3	14 × 14	1 × 1, 256; 3 × 1, 256; 1 × 3, 256; 1 × 1, 1024	5	
unit_3	14 × 14	1 × 1, 256; 3 × 1, 256; 1 × 3, 256/2; 1 × 1, 1024	1	
unit_4	7 × 7	1 × 1, 512; 3 × 1, 512; 1 × 3, 512; 1 × 1, 2048	2	
unit_4	7 × 7	1 × 1, 512; 3 × 1, 512; 1 × 3, 512/2; 1 × 1, 2048	1	
1 × 7 × 2	1 × 1	7 × 7 mean pool, 2048	1	
1 × 7 × 2	-	4 fc, Softmax	1	
ER Rule				1

**Table 3:** The correct rate of the model in the training set and test set.

Model	Training Set	Test Set
Multi-ResNet-67 + ER Rule	0.9916	0.9734
Wavelet packet + BP	0.9772	0.9646
Wavelet packet + RBF	0.9706	0.9553
Wavelet packet + SVM	0.9634	0.9521

## Data Availability

The data that support the findings of this study were derived from the following resources available in the public domain: [Case Western Reserve University Bearing Data Center website, available online at: https://engineering.case.edu/bearingdatacenter/welcome, accessed on 1 December 2025].

## Abbreviations

The following abbreviations are used in this manuscript:

ER Rule	Evidence Reasoning Rule
ResNet	Residual Network
STFT	Short-Time Fourier Transform
BP	Back Propagation network
RBF	Radial Basis Function network
SVM	Support Vector Machine
ES	Expert System
UKF	Unscented Kalman Filter
CFMDAS	Car Failure and Malfunction Diagnosis Assistance System
OLA	Online Approximator
ANN	Artificial Neural Network
EMD	Empirical Mode Decomposition
CWRU	Case Western Reserve University
HP	horsepower
RPM	Revolutions Per Minute
A-IBRB	automatic interval belief rule base
CMA-ES	Covariance Matrix Adaptation Evolutionary Strategies
